# Towards an automatic classification of protein structural domains based on structural similarity

**DOI:** 10.1186/1471-2105-9-74

**Published:** 2008-01-31

**Authors:** Vichetra Sam, Chin-Hsien Tai, Jean Garnier, Jean-Francois Gibrat, Byungkook Lee, Peter J Munson

**Affiliations:** 1 Mathematical and Statistical Computing Laboratory, DCB, CIT, NIH, DHHS, Bethesda, MD, USA; 2 Laboratory of Molecular Biology, CCR, NCI, NIH, DHHS, Bethesda, MD, USA; 3 Laboratoire de Mathematique Informatique et Genome, INRA, Jouy-en-Josas, France

## Abstract

**Background:**

Formal classification of a large collection of protein structures aids the understanding of evolutionary relationships among them. Classifications involving manual steps, such as SCOP and CATH, face the challenge of increasing volume of available structures. Automatic methods such as FSSP or Dali Domain Dictionary, yield divergent classifications, for reasons not yet fully investigated. One possible reason is that the pairwise similarity scores used in automatic classification do not adequately reflect the judgments made in manual classification. Another possibility is the difference between manual and automatic classification procedures. We explore the degree to which these two factors might affect the final classification.

**Results:**

We use DALI, SHEBA and VAST pairwise scores on the SCOP C class domains, to investigate a variety of hierarchical clustering procedures. The constructed dendrogram is cut in a variety of ways to produce a partition, which is compared to the SCOP fold classification.

Ward's method dendrograms led to partitions closest to the SCOP fold classification. Dendrogram- or tree-cutting strategies fell into four categories according to the similarity of resulting partitions to the SCOP fold partition. Two strategies which optimize similarity to SCOP, gave an average of 72% true positives rate (TPR), at a 1% false positive rate. Cutting the largest size cluster at each step gave an average of 61% TPR which was one of the best strategies not making use of prior knowledge of SCOP. Cutting the longest branch at each step produced one of the worst strategies.

We also developed a method to detect irreducible differences between the best possible automatic partitions and SCOP, regardless of the cutting strategy. These differences are substantial. Visual examination of hard-to-classify proteins confirms our previous finding, that global structural similarity of domains is not the only criterion used in the SCOP classification.

**Conclusion:**

Different clustering procedures give rise to different levels of agreement between automatic and manual protein classifications. None of the tested procedures completely eliminates the divergence between automatic and manual protein classifications. Achieving full agreement between these two approaches would apparently require additional information.

## Background

This work investigates a large number of different methods for producing an automatic classification of structural domains of proteins based on all-against-all pairwise structural similarity scores. We produce candidate classifications and compare them to the human expert-curated classification SCOP [1]. Perhaps the earliest attempt at automatic classification of protein structures was by Holm and Sander [2], who produced the FSSP database, consisting primarily of a tree obtained by applying hierarchical clustering using the pairwise structural similarity scores among a sequence representative set [3]. Later, Dietmann et al [4] refined FSSP by introducing the notion of domain [5]. More recent work by Gewehr *et *al. [6] and others [7-10], focused on the problem of assigning new, unclassified domains into their correct pre-established fold classes. Other authors [11-15] compared a set of pairwise similarity measures computed by various structure comparison methods to the SCOP (or other) pre-existing classification, to investigate the causes of divergence between automatically determined pairwise structural similarity and expert-curated classification. In particular, the comparison between FSSP, SCOP and CATH, by Hadley and Jones [12], makes use of FSSP pairwise scores between protein chains which are present in all three databases, as FSSP does not use domain as the classification unit. Although these studies advanced the understanding of divergence between automatic similarity and expert-curated classification, improvement of automatic classification procedures was not their primary objective.

Our earlier work [15] pointed to structural variations within SCOP folds as a main cause of divergence between automatic and expert-curated classifications. Intra-fold structural variation affects the measured structural similarity both within and between folds. The measured similarities are often not uniform among domains within a fold and the average similarity among domains within one fold can be different from that within another fold. We investigate different partitioning strategies in an attempt to accommodate such uneven similarity distributions. In particular, we investigate dendrogram cutting strategies as a potential means of isolating both tightly clustered, homogeneous folds and more heterogeneous ones, using a single procedure.

One source of potential confusion in the literature is the failure to explicitly consider the fundamental difference between mathematical properties of a similarity score matrix compared to a classification or partition. Mathematically, each can be represented as an M by M matrix where M is the number of protein domains. Unlike a pairwise similarity score matrix which can be any square matrix, a "partition matrix" is made of 0 or 1 elements indicating when two domains are in the same (1) or different (0) clusters of the partition. The partition matrix can always be transformed into a block-diagonal form by sorting the rows and columns appropriately, reflecting a property related to the definition of a partition itself. A partition of a set of domains is comprised of non-overlapping clusters, meaning that if A and B are in the same cluster, while B and C are also in the same cluster, then A and C are necessarily in that same cluster, a type of transitivity here called the "partition constraint". This property may be absent in a pairwise similarity matrix.

Building a classification by clustering based on pairwise similarity scores is essentially the same thing as transforming the pairwise similarity matrix into one which satisfies the partition constraint. Clearly, this process may force some domain pairs originally considered as dissimilar into the same cluster while other, similar pairs would be forced into different clusters. In many studies comparing the automatically determined pairwise similarities and a structural classification database [11-15], the effect of the presence of the partition constraint was not explicitly considered. Some of the reported discrepancy between similarity scores and classifications may potentially result from the failure of the similarity scores to satisfy this constraint. In the present study, we eliminate this factor by first converting the pairwise similarity dataset into a partition (via clustering) and then comparing the two partitions.

The derivation of a partition from a set of pairwise similarities is not a trivial process, but involves several distinct steps of computations, requiring careful analysis. Here, particular attention will be given to hierarchical clustering methods and dendrogram cutting strategies. We obtain a partition from pair-wise similarity scores by first transforming the similarity measure into a distance measure, applying various standard hierarchical clustering methods, obtaining a dendrogram, or binary cluster joining tree, and in the final step, applying a strategy which removes the root node and successively lower level nodes leaving behind a set of trees corresponding to the clusters in the partition. The pairwise similarity scores are provided by VAST [15,16], SHEBA [17] and DALI [18,19], three distinctive and efficient approaches for measuring structural similarity.

## Results

### Performance of hierarchical clustering as tree building methods

We generated pairwise similarity scores between all pairs of domains in the SCOP C class using three different structure alignment methods, VAST, SHEBA and DALI, and using two different similarity metrics for each method, altogether yielding 6 similarity matrices. We used four different hierarchical clustering methods (Single, Average, Complete linkage and Ward method [20]) to build dendrograms. The dendrograms were cut using seven SCOP-independent strategies and three SCOP-dependent strategies. Finally, two different criteria for terminating the tree cutting process were investigated. We stopped the cutting either when a 1% false positive rate (FPR) was reached or when the number of clusters matched the number of folds in the SCOP C class, which is 94 for the current dataset. The true positive rate at 1% false positive rate (TPR_01_) and the number of clusters in each partition, resulting from these various partitioning approaches, using the 1% FPR termination criterion, are reported in Tables 1 and 2.

**Table 1:** TPR values at 1% FPR (TPR_01_) for Hierarchical Cluster Methods and Tree Cutting Strategies.

Group^#^	Tree Cutting Strategy	Hierarchical Cluster Method	VAST Nres	VAST Pcli	SHEBA Nres	SHEBA Zscore	DALI Nres	DALI Zscore	Average	Range
2	Level	Average	28	24	23	33	28	38	29	15
2	Level	Complete	28	31	29	40	39	27	32	13
2	Level	Single	14	10	12	21	16	8	13	13
2	Level	Ward	50	48	48	54	50	49	50	6
3	Largest Size	Average	54	50	48	59	55	41	51	18
3	Largest Size	Complete	44	44	43	43	50	51	46	8
3	Largest Size	Single	29	22	27	39	34	13	27	26
3	Largest Size	Ward	61	59	61	58	62	65	61	7
3	Tree Completeness	Ward	57	59	61	56	56	63	58	7
3	Highest tree	Ward	57	61	62	56	57	63	59	7
1	Tree skewness	Ward	25	15	26	22	31	21	23	10
1	Longest branch	Ward	29	20	26	22	27	13	23	16
2	Maximum Entropy	Ward	45	40	43	46	58	48	46	18
1	BestTPR	Ward	12	13	13	10	8	16	12	8
4	Best ratio TPR &FPR	Ward	73	72	73	71	75	75	73	4
4	MI	Ward	73	71	72	71	76	76	73	5
	Direct pw^§^	none	50	52	60	61	52	67	57	17

^§^Direct pw is the direct pairwise comparison between pairwise similarity scores and SCOP folds in the C class, and does not involve clustering.

# the group to which the strategy belongs.

**Table 2:** Number of Clusters for Hierarchical Cluster Methods and Tree Cutting Strategies at 1%FPR.

Group	Tree Cutting Strategy	Hierarchical Cluster Method	VAST Nres	VAST Pcli	SHEBA Nres	SHEBA Zscore	DALI Nres	DALI Zscore
2	Level	Average	460	573	576	402	444	389
2	Level	Complete	307	250	332	195	221	396
2	Level	Single	810	1023	921	686	787	1041
2	Level	Ward	110	120	138	106	113	121
3	Largest Size	Average	183	210	222	190	169	256
3	Largest Size	Complete	81	84	88	94	69	82
3	Largest Size	Single	515	658	528	420	439	792
3	Largest Size	Ward	71	79	81	71	67	72
3	Tree Completeness	Ward	95	89	87	88	101	96
3	Highest tree	Ward	88	80	84	81	92	91
1	Tree skewness	Ward	603	701	603	592	606	641
1	Longest node	Ward	409	704	627	837	646	991
2	Maximum Entropy	Ward	143	146	109	101	81	109
1	BestTPR	Ward	1121	1112	1054	1106	1185	1029
4	Best ratio TPR &FPR	Ward	59	65	70	61	57	63
4	MI	Ward	105	76	72	68	55	77

Trees generated by Ward's method result in better performance. The TPR_01 _values obtained for Ward's clustering method are, with one exception, always higher than for the Average, Complete or Single linkage method. The simplest, level cut strategy with Ward's method achieves an average TPR_01 _of 50% across all six structure comparison scores. Complete or Average linkage clustering constitute the next best alternatives to Ward's method, with average TPR_01 _across all similarity scores and structure comparison methods, of 32% and 29% respectively. Single linkage clustering shows uniformly the most divergence from SCOP. Not only does Ward's method achieve the highest average TPR_01 _value, but the values vary less across different similarity scores and structure comparison methods, than do those for Complete, Average and Single Linkage, suggesting that Ward's is more satisfactory for this application.

With other tree cutting strategies the trend among various clustering methods is the same. For the largest size cut strategy, Ward's method gives an average TPR_01 _value of 61%, at least 10% better than for the three other clustering methods. Again, Complete and Average Linkage give similar average TPR_01 _values, while Single Linkage is very low in comparison. In view of the clearly superior results with Ward's method, we investigate tree cutting strategies using that method alone.

### Performance of tree cutting strategies

**Figure 1:**
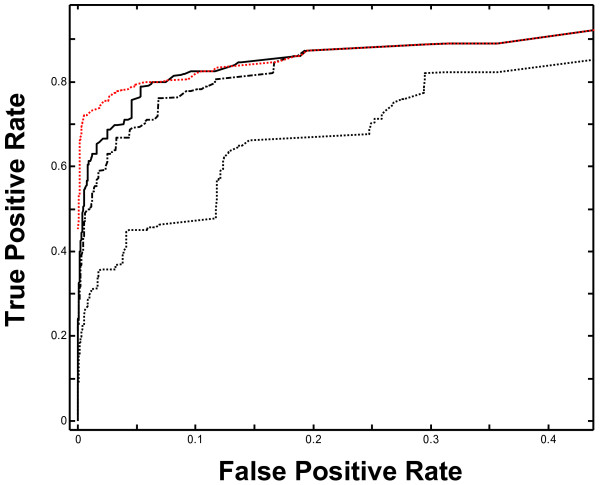
Receiver Operating Characteristic (ROC) curves for various tree cutting strategies, using the Ward's method clustering based on the VAST Nres similarity score. The true positive rate (TPR, Y axis) is plotted against the false positive rate (FPR, X axis). Black curves correspond to largest size cut (solid line), longest branch cut (dotted line) and level cut (dash-dot line) strategies, which use only of tree topology parameters. The red dotted curve corresponds to the MI cut strategy, which uses SCOP classification information directly, so is viewed only as an approximate upper bound to the SCOP-independent strategies. The Y-axis intercept for this curve is 0.45 and close to 0.0 for all SCOP-independent strategies. Only the portion of the curve for FPR values below 0.45 is shown. Between this FPR value and 0.63 relative positions of the curves do not vary. Above 0.63 FPR value all curve superpose to the MI cut curve.

In the following, the performance of tree-cutting strategies is analyzed. The 10 tested tree-cutting strategies fall into four groups according to their TPR_01 _values (Figure 1 and Table 1), and are represented by the longest branch cut (1), level cut (2), largest size cut (3) and mutual information cut (4) strategies. Figure 1 illustrates the comparative performance among these representative tree cutting strategies, for the VAST number of matched residues similarity score.

Among the four SCOP-independent tree cutting strategies shown, "largest size cut" achieves the best performance with its ROC curve dominating all other SCOP-independent ROC curves. Moreover, "largest size cut" ROC curve is the closest to that for Mutual Information cut, a strategy which attempts to maximize the agreement of the partition with SCOP at each step. Similar results (data not shown) were seen for SHEBA and DALI similarity scores.

The longest branch cut group (Table 1, Group 1) contains the strategies of lowest performance, e.g. tree skewness strategy, as indicated by their respective mean TPR_01 _values. Longest branch cut strategy results in one of the lowest agreements with SCOP, in particular for FPR values below 30% (Figure 1). Above this FPR level, its performance becomes more acceptable relatively to the upper bound given by the mutual information cut. This strategy seems to make more sense when inter-cluster distance is high, i.e. for highest nodes of the tree. But it behaves much worse than others when the inter-cluster distance is reduced, as its ROC curve is closer to the main diagonal than the ROC for other strategies. In the lowest part of the tree, inter-cluster distances vary only slightly so that the longest branch criterion for choosing among the many possibilities, the next sub-tree to be cut might not be discriminative enough.

Level cut and maximum entropy cut strategies form another group (Table 1, Group 2) characterized by middling performance. Their grouping is explained by the fact that these strategies perform comparably at low FPR values and their average TPR_01 _values are comparable. Higher variability among the TPR values for maximum entropy cut strategy is noted, however. Indeed, for VAST and SHEBA, level cut strategy is better than maximum entropy cut, as a rule, with DALI number of matched residues (Nres) score the only exception. The highest TPR_01 _for the maximum entropy cut strategy (58%) is obtained with the Nres metric, while the lowest value (40%) is obtained by VAST Pcli.

The third, largest size cut strategy group (Table 1, Group 3) also includes the tree completeness cut, and highest tree cut strategies and corresponds to SCOP-independent strategies of highest performance, with average TPR_01 _values ranging from 58 to 61%. The tree height and tree completeness are closely related to the number of leaves, i.e. the size of the tree. These results indicate that of these three topological properties, the size is a better measure, for the purpose of creating partitions that agree with SCOP. This is true for partitions of small size (the large FPR values), but also when the number of clusters is large (the small FPR values).

The fourth group (Table 1, Group 4) is made up of mutual information cut and Best TPR/FPR Ratio cut strategies, all of which make direct use of the SCOP fold partition and thus are not useful for an independent classification. The poor performance of the best TPR cut strategy (Table 1, Group 1) indicates that strategies which ignore the false positives will not generate SCOP-like partitions.

The size of each partition is reported in Table 2, and shows a large variation in the number of clusters depending on the hierarchical clustering method. Single linkage always leads to partitions with a high number of clusters, in fact close to the total number of domains M, when FPR is kept low at 1%.

### Comparison of partitions

The relative organization of automatically generated partitions can be understood by first finding a distance measure between two partitions and then displaying the partitions in a distance preserving graph. The distance between partitions (Eq. 8), computed as the total number of disagreements about whether or not a pair of domains is in the same cluster, is reported in the Table 3. In this exercise, partitions with exactly 94 clusters were compared, including the SCOP fold partition.

**Table 3:** Distance between partitions*, (Δ-distance in 1000s)

Partition	VNS	SNS	DNS	VPS	SZS	DZS	VNL	SNL	DNL	VPL	SZL	DZL	SCOP
VNS	0	30	24	31	30	28	27	46	37	47	41	51	65
SNS	30	0	24	35	25	23	44	32	38	51	38	51	65
DNS	24	24	0	31	21	22	39	42	25	48	37	48	66
VPS	31	35	31	0	37	32	43	50	43	28	47	56	71
SZS	30	25	21	37	0	27	42	43	37	50	28	51	69
DZS	28	23	22	32	27	0	44	47	36	49	42	41	63
VNL	27	44	39	43	42	44	0	48	37	49	37	51	75
SNL	46	32	42	50	43	47	48	0	41	56	47	49	84
DNL	37	38	25	43	37	36	37	41	0	52	39	53	73
VPL	47	51	48	28	50	49	49	56	52	0	54	56	83
SZL	41	38	37	47	28	42	37	47	39	54	0	56	70
DZL	51	51	48	56	51	41	51	49	53	56	56	0	91
SCOP	65	65	66	71	69	63	75	84	73	83	70	91	0

*Partitions obtained from automatic methods, are identified by three letter code. The first letter in the name of each automatic partitions indicates the structures comparison method (V, S and D, for VAST, SHEBA and DALI respectively), the second letter, the score (N, P, and Z, for number of matched residues, Pcli and Zscore, respectively), and the third letter indicates the level cut (L) or largest size cut (S) strategies. SCOP corresponds to the C class fold partition of the manual classification SCOP.

Average distances (Table 4) among partitions within a given comparison method (either VAST, SHEBA or DALI), are uniformly lower than distances between these latter and SCOP, by almost a factor two. Partitions from VAST tend to be slightly more heterogeneous than those from DALI or SHEBA. On average, automatic partitions from the three comparison methods are similarly distant from SCOP. Similarly, for a given cut strategy, the average distance among its partitions is consistently smaller than the distances from those automated partitions to SCOP (Table 4). Further, largest size cut partitions are half as distant among themselves as are those of level cut strategy. The pattern of distances confirm that the largest size cut partitions are much closer to the SCOP fold partition than level cut partitions, as also seen using the ROC curves.

**Table 4:** Δ-distance*, in 1000s, between partitions within each method and minimum Δ-distance from each method to SCOP

Method	Average Distance◊	Maximum Distance◊	Average Distance to SCOP	Minimum Distance to SCOP
1 VAST	43	56	73	65
2 SHEBA	35	47	72	65
3 DALI	37	53	73	63
4 Size	28	37	66	63
5 Level	48	59	80	70

*Δ-distance as defined in Method, Eq. 8.

◊ Average or Maximum distance among partitions of a particular structural comparison program, or of a tree-cutting strategy. Average distance among SHEBA partitions for example, is obtained by averaging across all SHEBA similarity scores and tree cutting strategies. Average distance among largest size cut partitions for example, is obtained by averaging across all similarity scores of structure comparison programs VAST, SHEBA and DALI.

Table 5 shows that every automatic partition is much closer to other automatic partitions, than to the SCOP fold partition. For example, the DALI Z-score level-cut partition (DZL) is at most 56,000 units from all other automated partitions, but 91,000 units from SCOP. The closest automated partition to SCOP is DALI Z-score, largest size cut (DZS), with a distance of 63,000 units, which in turn is no farther than 49,000 units from all other automated partitions. DZL is farthest from both SCOP and from at least one other automatic partition. Conversely, DALI Nres with Largest size-cut (DNS) is perhaps most representative of automated methods as it minimizes the maximum distance to other such methods.

**Table 5:** Distance between Automatic Partitions and SCOP fold Partition

Automatic Partition	Maximum Δ-distance to Others*	Δ-distance to SCOP*	FPR (%)	TPR (%)
**VNS**	51	65	0.5	53
**SNS**	51	65	0.7	59
**DNS**	48	66	0.5	54
**VPS**	56	71	0.8	55
**SZS**	51	69	0.5	51
**DZS**	49	63	0.6	60
**VNL**	51	75	1.5	54
**SNL**	56	84	1.6	55
**DNL**	53	73	1.1	56
**VPL**	56	83	1.6	53
**SZL**	56	70	1.1	56
**DZL**	56	91	1.9	54

Automatic partitions are identified by three letter code used in table 3.

"Others" refers to other automatic partitions.

*Δ-distance, as defined in Methods, in 1000s.

FPR (%) – False Positive Rate (Methods, *Eq*. 4)

TPR (%) – True Positive Rate (Methods, *Eq*. 6)

**Figure 2:**
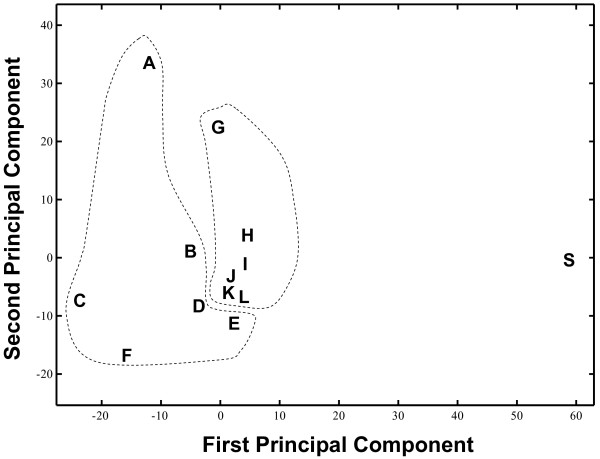
Classical multidimensional scaling (MDS) plot of various automatically generated partitions and the SCOP fold partition. The MDS plot approximately preserves distances, Δ, between partitions (see Methods). The X axis represents the projection of the location of each partition onto the first eigenvector (first principal component), while the Y axis is a projection onto the second eigenvector (second principal component). The X and Y axes of the plot are scaled to represent the distance, Δ, divided by 1000. Automatic partitions are obtained with Ward's method clustering, based on two different similarity score for each of the three methods VAST, SHEBA and DALI. Each partition is designated by an uppercase letter: A, VAST Pcli Level cut; B, VAST Nres Level cut; C, DALI Zscore Level cut; D, DALI Nres Level cut; E, SHEBA Zscore Level cut; F, SHEBA Nres Level cut; G, VAST Pcli Largest Size cut; H, VAST Nres Largest Size cut; I, DALI Zscore Largest Size cut; J, DALI Nres Largest Size cut; K, SHEBA Zscore Largest Size cut; L, SHEBA Nres Largest Size cut, and S, the expert-curated partition SCOP. Automatic partitions resulting from the same tree-cutting strategy, are grouped together within the same dotted area.

A convenient, intuitive representation of the organization of these partitions is obtained using multi-dimensional scaling, a technique which embeds the 13 partitions into a low-dimension Euclidean space so that the pairwise distances are approximately preserved. Figure 2 represents the automatic and SCOP fold partitions in a 2-dimensional space. Automatic partitions are well-separated from the SCOP fold partition, which appears isolated. Largest size-cut partitions are generally closer to SCOP than are level-cut partitions, and are also less spread.

### Comparison of cluster size distributions

**Figure 3:**
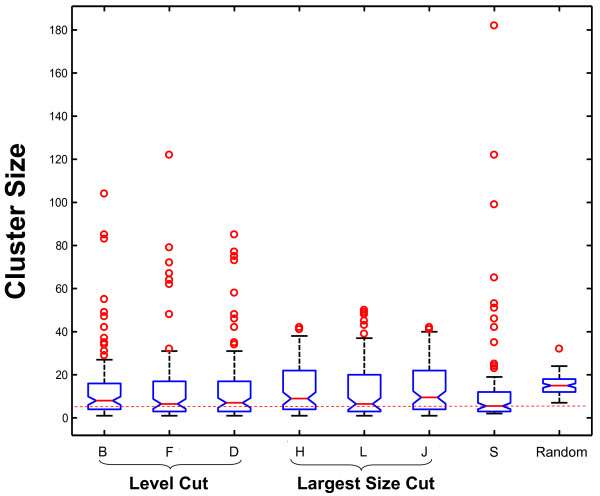
Box plots of the cluster size distributions for six automatically generated partitions with 94 clusters and the SCOP partition of the C class. Each partition is associated with a box plot. A box plot summarizes the following statistics: median (line within the box), upper and lower quartiles (the upper and lower hinge of the box respectively), minimum and maximum data values (the ends of the vertical dash lines), and outliers (circles). For comparison, a "random" partition would have cluster size following approximately a Poisson distribution with intensity parameter equal to M/94 ~ = 14 domains per cluster. The partitions are labelled as explained in Figure 2. The SCOP partition and Random partitions are identified. All distributions necessarily have the same mean value, M/94, but show differing medians, interquartile ranges, tail lengths and maximal values. Both the SCOP and Random distributions show minimum values substantially above the automated partitions. The horizontal red dash line indicates the median value of SCOP fold partition.

In addition to the number of clusters in a partition, the distribution of cluster sizes may be of interest in selecting an appropriate classification. Figure 3 shows the cluster size distribution for six automatically generated partitions and for SCOP. For comparison, a partition of the same number of domains randomly assigned to 94 clusters with equal probability is shown. This distribution was approximated by a Poisson distribution with a mean value of 1330/94 ≅ 14.15.

We observe first that all partitions, including SCOP, have lower median cluster size and greater spread of size than for random. There is evidently sufficient signal strength within the similarity score matrix to influence the size distribution. Second, the 75th percentiles for automated methods tend to be larger than for SCOP, while the SCOP distribution shows larger positive skewness, with a greater number of unusually large clusters. Third, there is some uniformity in the size distribution within tree cutting strategies, with largest size cut showing somewhat higher 75th percentiles and less skewness than do the level cut distributions. Level cut distributions are closer the SCOP distribution in terms of median, 75th percentile size and the larger number of outliers, than the largest size cut distributions.

The largest SCOP fold (c.1), is in fact, split by all strategies and methods as none include a cluster with 182 domains. Largest size cut strategy intentionally eliminates outliers of large size, thereby creating more clusters of intermediate sizes, with greater spread (inter-quartile range) than for level cut. The level cut generates a few large size outliers but the clusters are smaller, typically. This is consistent with the observation made earlier that the level cut behaves like the maximum entropy cut at small FPR ranges. Using partitions of 94 clusters, we find FPR values higher than 1% for level cut strategy but lower than 1% for largest size cut strategy (Table 5), indicating that a trade-off must be made in matching the size distribution as well as maximizing the TPR in relationship to the SCOP partition.

### Identification of dispersed folds

The spanning cluster of a SCOP fold is the smallest cluster in the dendrogram which spans or includes all domains in that fold. The excess span of that fold (see Methods) are the domains from other folds that are included in its spanning cluster. A homogeneous cluster is a cluster which includes only domains from a single SCOP fold. The size of the excess span and the size of the largest homogeneous cluster are given in Table 6 for each fold in SCOP C Class for three different dendrograms. These two measures allow comparison of each dendrogram to SCOP on a fold by fold basis, and can highlight regions of agreement or disagreement between the two systems. When the excess span is zero and the largest homogenous cluster is 100% of the fold size, the dendrogram and SCOP are in perfect agreement for that fold. A hypothetical tree-cutting strategy could potentially isolate this particular sub-tree to form a cluster exactly matching that fold.

**Table 6:** Excess Span and Largest Homogeneous Cluster Size for SCOP C Class folds.

					**Excess Span Size^§^**				**Largest Homog. Cluster (%) ^+^**			
**Row**	**fold**	**SCOP fold description**	Ndom*	**Nfam**^#^	**VAST**	**SHEBA**	**DALI**	**INT◊ **	**VAST**	**SHEBA**	**DALI**	**Nc**^$^

		CONSISTENT FOLDS										
1	c.100	Thiamin pyrophosphokinase, catalytic domain	2	1	0	0	0	0	100	100	100	1
2	c.101	Undecaprenyl diphosphate synthase	2	1	0	0	0	0	100	100	100	1
3	c.106	SurE-like	2	1	0	0	0	0	100	100	100	1
4	c.109	PEP carboxykinase N-terminal domain	4	1	0	0	0	0	100	100	100	1
5	c.114	YchN-like	2	1	0	0	0	0	100	100	100	1
6	c.117	Amidase signature (AS) enzymes	3	1	0	0	0	0	100	100	100	1
7	c.16	Lumazine synthase	3	1	0	0	0	0	100	100	100	1
8	c.17	Caspase-like	3	2	0	0	0	0	100	100	100	1
9	c.22	Ribosomal protein L4	2	1	0	0	0	0	100	100	100	1
10	c.27	Nucleoside phosphorylase/phosphoribosyltransferase catalytic domain	3	1	0	0	0	0	100	100	100	1
11	c.28	Cryptochrome/photolyase, N-terminal domain	4	1	0	0	0	0	100	100	100	1
12	c.32	Tubulin, GTPase domain	2	1	0	0	0	0	100	100	100	1
13	c.33	Cysteine hydrolase	3	2	0	0	0	0	100	100	100	1
14	c.34	DFP DNA/pantothenate metabolism flavoprotein	2	1	0	0	0	0	100	100	100	1
15	c.36	Thiamin diphosphate-binding fold (THDP-binding)	19	4	0	0	0	0	100	100	100	1
16	c.39	Nicotinate mononucleotide:5,6-dimethylbenzimidazole phosphoribosyltransferase (CobT)	2	1	0	0	0	0	100	100	100	1
17	c.42	Arginase/deacetylase	3	2	0	0	0	0	100	100	100	1
18	c.43	CoA-dependent acyltransferases	7	3	0	0	0	0	100	100	100	1
19	c.5	MurCD N-terminal domain	2	1	0	0	0	0	100	100	100	1
20	c.50	Leucine aminopeptidase (Aminopeptidase A), N-terminal domain	2	1	0	0	0	0	100	100	100	1
21	c.59	MurD-like peptide ligases, peptide-binding domain	5	2	0	0	0	0	100	100	100	1
22	c.6	Cellulases	3	1	0	0	0	0	100	100	100	1
23	c.62	vWA-like	8	2	0	0	0	0	100	100	100	1
24	c.65	Formyltransferase	3	1	0	0	0	0	100	100	100	1
25	c.67	PLP-dependent transferases	35	6	0	0	0	0	100	100	100	1
26	c.68	Nucleotide-diphospho-sugar transferases	15	12	0	0	0	0	100	100	100	1
27	c.7	PFL-like glycyl radical enzymes	4	4	0	0	0	0	100	100	100	1
28	c.70	Nucleoside hydrolase	2	1	0	0	0	0	100	100	100	1
29	c.71	Dihydrofolate reductases	8	1	0	0	0	0	100	100	100	1
30	c.73	Carbamate kinase-like	2	2	0	0	0	0	100	100	100	1
31	c.74	AraD-like aldolase/epimerase	3	1	0	0	0	0	100	100	100	1
32	c.76	Alkaline phosphatase-like	6	3	0	0	0	0	100	100	100	1
33	c.77	Isocitrate/Isopropylmalate dehydrogenases	4	2	0	0	0	0	100	100	100	1
34	c.79	Tryptophan synthase beta subunit-like PLP-dependent enzymes	6	1	0	0	0	0	100	100	100	1
35	c.81	Formate dehydrogenase/DMSO reductase, domains 1–3	5	1	0	0	0	0	100	100	100	1
36	c.83	Aconitase iron-sulfur domain	2	1	0	0	0	0	100	100	100	1
37	c.86	Phosphoglycerate kinase	2	1	0	0	0	0	100	100	100	1
38	c.89	Phosphofructokinase	2	1	0	0	0	0	100	100	100	1

		MODERATELY CONSISTENT FOLDS										
39	c.25	Ferredoxin reductase-like, C-terminal NADP-linked domain	12	5	643	0	0	0	92	100	100	1
40	c.61	PRTase-like	17	2	0	0	68	0	100	100	88	1
41	c.82	ALDH-like	7	2	0	954	0	0	100	86	100	1
42	c.87	UDP-Glycosyltransferase/glycogen phosphorylase	6	6	0	937	0	0	100	83	100	1
43	c.116	alpha/beta knot	5	3	3	0	0	0	80	100	100	1
44	c.41	Subtilisin-like	4	2	0	0	1056	0	100	100	75	1
45	c.98	MurF and HprK N-domain-like	4	2	8	0	0	0	75	100	100	1
46	c.46	Rhodanese/Cell cycle control phosphatase	7	3	130	54	0	0	86	86	100	1
47	c.57	Molybdenum cofactor biosynthesis proteins	3	2	1056	0	0	0	67	100	100	1
48	c.93	Periplasmic binding protein-like I	13	1	3	0	0	0	62	100	100	1
49	c.45	(Phosphotyrosine protein) phosphatases II	12	2	0	0	267	0	100	100	58	1
50	c.107	DHH phosphoesterases	2	2	0	0	1058	0	100	100	50	1
51	c.18	DNA glycosylase	2	2	1057	0	0	0	50	100	100	1
52	c.108	HAD-like	11	10	1048	932	0	0	55	91	100	1
53	c.10	Leucine-rich repeat, LRR (right-handed beta-alpha superhelix)	18	10	304	0	0	0	44	100	100	1
54	c.48	TK C-terminal domain-like	5	3	50	0	47	0	60	100	80	1
55	c.14	ClpP/crotonase	11	3	240	335	0	0	73	64	100	1
56	c.24	Methylglyoxal synthase-like	3	3	450	0	27	0	67	100	67	1
57	c.8	The "swivelling" beta/beta/alpha domain	10	8	0	28	4	0	100	80	40	1
58	c.63	CoA transferase	5	2	0	1	1	0	100	60	60	1
59	c.55	Ribonuclease H-like motif	53	19	400	362	0	0	94	21	100	1
60	c.91	PEP carboxykinase-like	4	2	449	0	80	0	50	100	50	1
61	c.95	Thiolase-like	13	2	4	0	4	0	46	100	46	1

		DISPERSED FOLDS										
62	c.47	Thioredoxin fold	51	13	1	1	1	0	45	82	82	2
63	c.12	Ribosomal proteins L15p and L18e	2	1	1	3	1	0	50	50	50	2
64	c.31	DHS-like NAD/FAD-binding domain	10	5	4	3	4	3	70	70	70	2
65	c.1	TIM beta/alpha-barrel	182	70	3	1148	1148	3	53	43	62	6
66	c.15	BRCT domain	7	4	130	12	4	2	71	86	86	2
67	c.13	SpoIIaa-like	3	2	10	4	7	3	67	67	67	2
68	c.30	PreATP-grasp domain	11	5	4	6	7	3	64	36	45	3
69	c.51	Anticodon-binding domain-like	13	6	6	35	7	0	54	69	77	2
70	c.3	FAD/NAD(P)-binding domain	46	5	7	1015	1014	7	41	41	41	8
71	c.84	Phosphoglucomutase, first 3 domains	6	1	10	94	266	10	67	67	67	2
72	c.4	Nucleotide-binding domain	7	3	46	18	14	14	57	29	29	3
73	c.9	Barstar-like	2	2	18	413	593	17	50	50	50	2
74	c.19	FabD/lysophospholipase-like	3	2	1056	940	40	40	67	67	67	2
75	c.53	Resolvase-like	9	3	444	44	65	16	44	44	44	3
76	c.97	Cytidine deaminase-like	4	2	45	411	139	28	75	75	75	2
77	c.44	Phosphotyrosine protein phosphatases I-like	3	2	52	57	120	46	67	67	67	2
78	c.58	Aminoacid dehydrogenase-like, N-terminal domain	11	5	72	404	59	32	55	55	55	3
79	c.49	Pyruvate kinase C-terminal domain-like	6	2	237	94	182	61	67	67	67	2
80	c.52	Restriction endonuclease-like	23	21	1036	216	141	128	91	57	91	3
81	c.72	Ribokinase-like	15	7	185	372	165	151	67	67	67	2
82	c.80	SIS domain	6	3	198	420	1054	23	67	67	67	2
83	c.92	Chelatase-like	11	5	311	932	221	200	36	36	36	3
84	c.60	Phosphoglycerate mutase-like	7	4	446	415	280	237	57	57	57	2
85	c.26	Adenine nucleotide alpha hydrolase-like	42	11	1017	913	460	459	29	29	29	7
86	c.94	Periplasmic binding protein-like II	25	2	654	970	592	511	96	84	88	2
87	c.66	S-adenosyl-L-methionine-dependent methyltransferases	35	23	620	908	1025	602	23	80	83	3
88	c.78	ATC-like	12	2	643	931	1048	625	42	33	58	3
89	c.35	Phosphosugar isomerase	3	2	652	940	1057	634	67	67	67	2
90	c.37	P-loop containing nucleotide triphosphate hydrolases	122	20	937	821	938	814	25	40	28	9
91	c.23	Flavodoxin-like	65	31	1045	878	1046	873	14	18	18	18
92	c.56	Phosphorylase/hydrolase-like	24	11	1035	919	1036	912	75	83	75	4
93	c.2	NAD(P)-binding Rossmann-fold domains	99	10	960	928	961	921	24	32	32	9
94	c.69	alpha/beta-Hydrolases	51	26	1059	1064	1060	1059	35	96	35	2

Ward Hierarchical Cluster Tree generated using Nres metric for VAST, SHEBA and DALI.

*Ndom, the number of domains in the fold.

^#^Nfam, the number of families in the fold.

^§^Excess Span Size is Span less Ndom; Span is the number of domains contained in the smallest cluster containing all domains of the fold.

◊ INT is the size of the intersection of the excess spans for VAST, SHEBA and DALI.

^+^Largest Homog. Cluster is the ratio of the size of the largest homogeneous cluster of the fold to the size of the fold itself, expressed as percent.

^$^Nc is the minimum number of homogeneous clusters in the fold, across VAST, SHEBA and DALI.

Table is sorted in ascending order of minimum excess span size, then in the ascending order of INT, and then by the average Largest Homog. Cluster Size value for the three methods in the descending order.

The first 38 folds reported in Table 6 are in perfect agreement with dendrograms of all three structure comparison methods VAST, SHEBA and DALI. Together they comprise 187 of 1330 domains in the C-class. The next 23 folds (Table 6, rows 39–61, comprising 227 domains) agree perfectly with the dendrogram of at least one structure comparison method. Thus 61 out of 94 SCOP folds in the C class are consistent with the dendrogram built from at least one pairwise structural similarity measure.

Structure comparison methods differ in the consistency of their associated dendrogram with the SCOP folds. Dendrogram derived from each similarity score method disagree with SCOP for various folds. Forty-eight folds (comprising 1080 domains) disagree for the VAST tree, 41(1026 domains) for the SHEBA tree, and 43 (991 domains) for the DALI tree, according to this criterion.

We consider a fold to be highly dispersed if it disagreed with trees of all three structure comparison methods. There are 33 such folds (comprising 916 domains) and they are reported in Table 6, row 62–94. None of these 33 folds could be obtained as a homogeneous cluster, thus each contributes to the loss of agreement between any automatic partition and the SCOP fold partition. For these 33 folds, the intersection of their excess span was computed, and reported in Table 4, column labelled INT. INT is a measure of the disagreement with SCOP that remains even if all three dendrograms were combined. Thirty-one out of 33 folds give rise to a positive INT, meaning that same domains contributed to their dispersion within trees. These 31 folds therefore contribute to the remaining distance between the automatic and expert-curated partitions, regardless of tree cutting strategy or structure comparison method used.

### Dispersion caused by low structural similarity within folds

**Figure 4:**
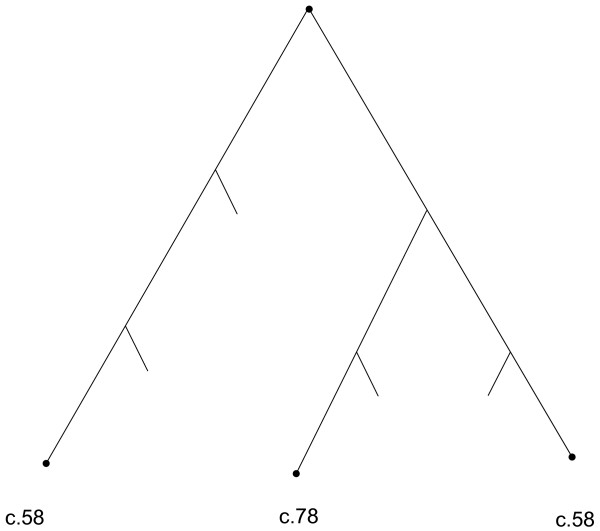
Schematic of dispersion of fold c.58. Nodes labelled c.58 represent two of the homogeneous clusters of fold c.58. Node labelled c.78 is one homogeneous cluster of fold c.78. Relative join positions reflected from the complete Ward's method dendrograms, based on clustering the Nres similarity scores.

To examine such dispersed folds in detail, we select two examples. Figure 4 schematically represents the situation of fold c.58 within each dendrogram. Instead of forming one homogeneous cluster including all domains of the fold, c.58 consists of mainly two homogeneous clusters set far apart in the tree. Between these two homogeneous clusters, domains from other C class folds intervene, in particular from c.78. Thus, one homogeneous cluster of fold c.58 is considered more similar to domains from fold c.78 than to other domains from fold c.58, and this situation pertains to all three structure comparison methods.

**Figure 5:**
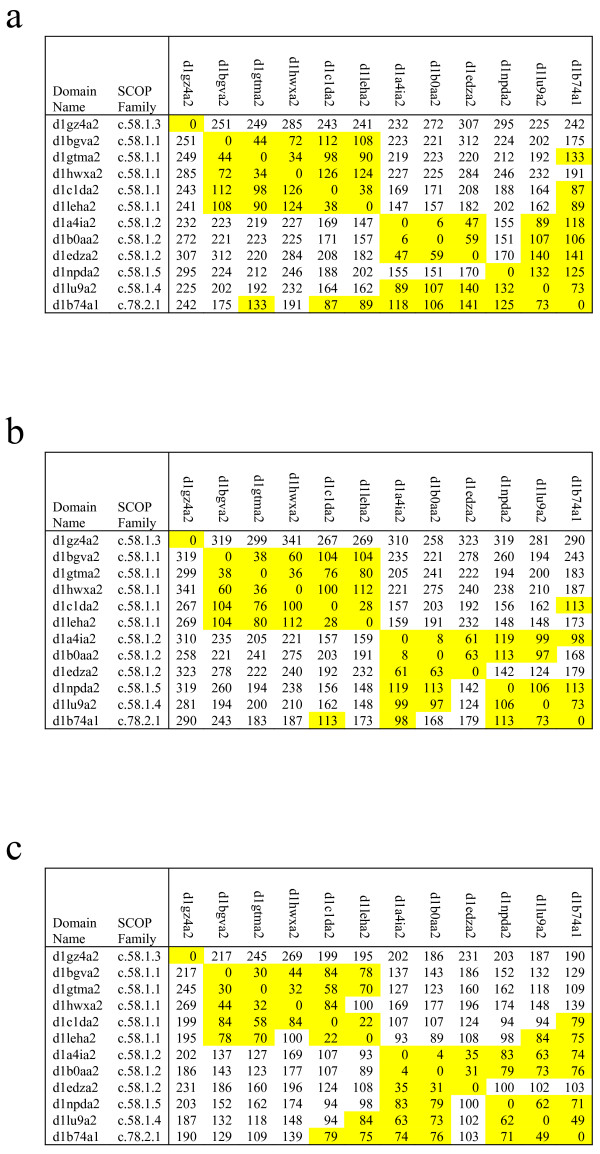
Matrices of pairwise distances among domains of folds c.58 and c.78. Pairwise distances between domains were obtained from the pairwise similarity scores number of matched residues, according to Eq(1) and symmetrized as specified in Methods. The yellow color corresponds to pairwise distances smaller than141, 119 and 88 for VAST matrix (a), SHEBA matrix (b) and DALI matrix(c) respectively. The white color corresponds to pairwise distances higher than 141, 129 and 88 for (a), (b) and (c) respectively.

Figure 5a, b and 5c presents the matrices of pairwise distances between all domains within folds c.58, for VAST, SHEBA and DALI, respectively. Coding the distances by color makes obvious why fold c.58 is split into mainly two homogeneous clusters by all three dendrograms. Figure 5a, b and 5c also include domain d1b74a1 from fold c.78, which is highly similar to domains of fold c.58. Indeed, many of the pairwise distances involving d1b74a1 (coded yellow), are small enough that it would fit into any cluster of fold c.58. This pattern of high distance between homogeneous clusters of c.58 and low distance of a fold c.78 domain to members of c.58 explains why no clustering method and no tree-cutting strategy is likely to perfectly identify fold c.58.

**Figure 6:**
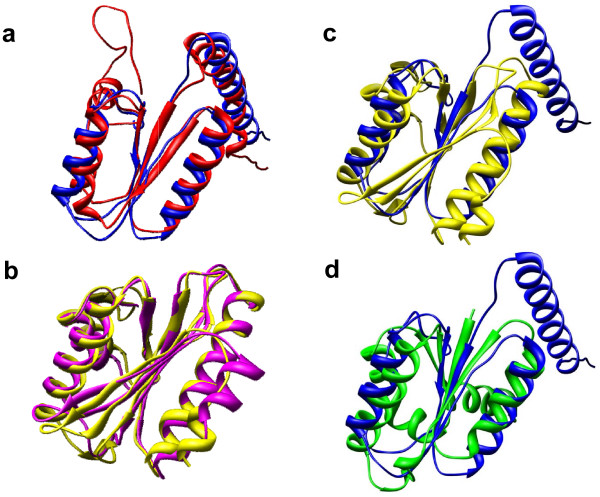
Superposition of c.58 and c.78 domain pairs, by DALI alignment. **Panel (a)**: one intra-cluster, cluster 1, pair of c.58, d1edza2 (146 residues, red) and d1b0aa2 (121 residues, blue), 118 residues aligned, RMSD = 2.7Å, and Zscore = 14.4. **Panel (b)**: another intra-cluster, cluster 2, pair of c.58, d1c1da2 (148 residues, yellow) and d1leha2 (134 residues, magenta), 130 residues aligned, RMSD = 1.4Å and Zscore = 21.3. **Panel (c)**: inter-cluster pair of c.58, between cluster 1 and cluster 2, d1b0aa2 (blue) and d1c1da2 (yellow), 81 residues aligned, RMSD = 4.7Å and Zscore = 4.5. **Panel (d)**: inter-fold domain pair between d1b74a1 (c.78, 105 residues, green) and d1b0aa2 (c.58, 121 residue, blue), 75 residues aligned, RMSD = 3.3Å, Zscore = 6.2. Both c.58 and c.78 folds are characterized by having a core of 3 layers a/b/a with a parallel beta-sheet of 4 strands, ordered 2134. Under hierarchical clustering, fold c.58 is comprised of two homogeneous clusters (Figure 4).

This pattern of structural similarity measures is further analyzed based on structural superposition of domains. Figure 6 (a) and 6 (b) superpose domain pairs from the same homogeneous cluster, and show the degree of structural similarity typical within cluster. The structural superposition of the domains from different homogeneous clusters of c.58 is shown in Figure 6 (c). The low similarity observed here is mainly due to the difference of the N terminal features, with the 3 layer a/b/a feature typical of this fold present in both domains. Figure 6 (d) is the structural superposition of a domain from c.58 with one from c.78. Again, the strands and helices making the 3 layer a/b/a feature are well aligned.

In Figure 6, the structural superposition (d) of domains from different folds produces even a better alignment than the superposition (c) of domains from two different clusters within the same c.58 fold. The disagreement between automatic and expert-curated classification, arises directly from the low similarity within fold c.58, and the substantial similarity to domains of another fold. It becomes impossible to merge the two distinct homogeneous clusters of fold c.58 without including domains from c.78 as well.

**Figure 7:**
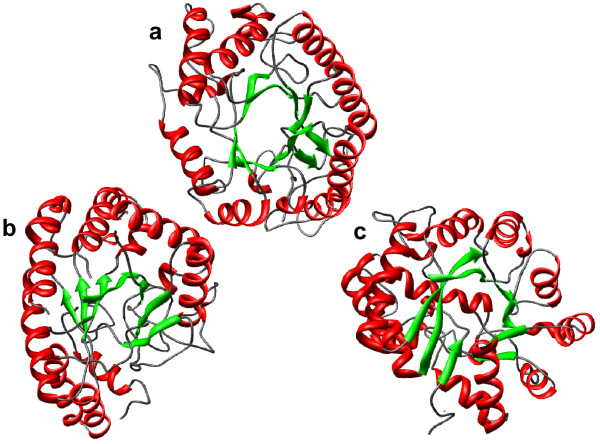
Structures from TIM barrel fold c.1 and its variant fold c.6. **Panel (a): **d1clxa_, a typical TIM Barrel domain belonging to family c.1.8.3 with 345 residues. **Panel (b)**: d1dysa_ c.6.1.1 with 345 residues. **Panel (c)**: d1a4ma_, also a TIM Barrel, belonging to family c.1.9.1 with 349 residues. These domains belong to three different homogeneous clusters, by all three programs. The pairwise distances by VAST, SHEBA and DALI, between structures (a) and (c) are (334*, 440, 318), between (b) and (a) are (236, 350, 256), and between (b) and (c) are (420, 500, 344). The pairwise number of matched residues (Nres) by VAST, SHEBA and DALI between structures (a) and (c) are (180*, 127, 188), between (a) and (b) are (227, 170, 217) and between (b) and (c) are (137, 97, 175). *This corresponds to the best alignment of the two non symmetric pairwise alignments produced by VAST.

As another example, we select fold c.1, one of the largest in the dataset, and a highly dispersed fold. The intersection of spanning clusters for this fold includes three domains from the single fold c.6, Cellulases, which are partial barrels. Fold c.6 is found to be easily identifiable by all three similarity methods (Table 6, row 22). Figure 7 shows two TIM barrel fold structures and one Cellulases structure. TIM barrel structures are easily recognizable and are not likely to be confounded with any other folds by the human expert. The typical TIM barrel structure d1clxa_ (Figure 7 a) appears to be more similar to the Cellulases structure d1dysa_ (Figure 7 b), than to another TIM barrel, d1a4ma_ (Figure 7 c), by all three structure comparison methods. TIM barrel structure (c) has 8 strands although two of them are much longer and two are much shorter than the rest making the barrel somewhat distorted, compared to the typical TIM barrel structure (a). The lower similarity between a typical TIM barrel domain and another member of that fold, compared to the similarity of a c.6 domain to the typical TIM barrel, means that automated clustering methods cannot separate these two folds perfectly. The structural distinctions between the two folds are evidently too subtle to be detected by these structure comparison methods.

## Discussion

There are two approaches for comparing results from automatic structure comparison methods to an expert-curated reference classification such as SCOP. One can either directly compare the pairwise structural similarity measures to a similar measure derived from the reference partition, or produce a partition from the pairwise structural similarity score and compare it to the reference partition. We adopted the latter approach in this study, as we expected that enforcing partitioning constraints would introduce a new element that is not present in the pairwise similarity measures alone.

We explored a variety of methods for obtaining automatic partitions from pairwise structural similarity measures computed by VAST, SHEBA and DALI. Specifically, four different hierarchical clustering methods were used to construct dendrograms or binary trees, which were then cut into sub-trees by ten different tree-cutting strategies, to produce partitions. The results show that the combination of Ward's method with the largest size cut strategy has best agreement with SCOP among all combinations of clustering methods and tree-cutting strategies explored so far.

Trees generated by Ward's method result in partitions agreeing better with SCOP folds, than those generated by Single, Complete or Average linkage, regardless of the tree cutting strategy applied. Clusters formed by Ward's method tend to be highly concentrated around a mean, as they are formed so that the variance within the cluster is minimized. This suggests that SCOP folds are constructed based on the cohesiveness of the group as a whole rather than on similarity of individual pairs. This may be a more appropriate view of folds than as complete sub-graphs where every structure is related to every other structure as suggested by Complete linkage clustering. Although Average linkage produces clusters which are organized around a constructed mean, it does not minimize the variance around this mean, in contrast to Ward's method. Its lower performance relative to Ward's method strengthens the view of folds as structurally cohesive groups of domains. The mediocre results obtained by Single linkage emphasize even more the suitability of the notion of structural cohesiveness in modelling folds. Indeed Single linkage folds can be seen as minimum spanning trees, which do not necessarily require structural cohesiveness.

Largest size tree cutting strategy and those associated with it, such as highest tree cut and tree completeness cut, achieve much better agreement with SCOP, than other SCOP independent strategies. In particular they out-perform the "standard" level cut strategy. This is an unexpected result and suggests that the fold size was perhaps an implicit criterion, among others, in the formation of SCOP folds and that large groups of protein structures were split even when their internal similarity was higher than the internal similarity of smaller sized group of proteins. The small spread of the sizes of SCOP folds shown in Figure 3 supports this notion. On the other hand, a highly structurally distinctive fold such as TIM barrel (c.1) which is highly populated relative to the majority of folds and easily, visually recognizable, form an exceptionally large cluster.

The best combination of hierarchical clustering and SCOP independent tree cutting strategy e.g. Ward's method with either largest size, tree completeness or highest tree cut, resulted in an average TPR_01 _values (61%, 58% and 59% respectively) that are only slightly above the average agreement of 57% achieved by comparing pairwise similarities directly, without clustering, to SCOP folds partition, using the same set of SCOP C class domains. This suggests the idea that the persistent discrepancy between automatically determined similarity and SCOP is most likely not due to the partitioning constraint, even though all possible partitioning strategies, such as most recently developed clustering techniques in [21,22] for example, have not been examined. Figure 2 provides additional evidence supporting this view. It shows that automatic partitions produced by introducing partitioning constraints into pairwise structural similarity measures, cluster together in the space of partitions, far away from the expert-curated classification SCOP implying that automatic structure comparison methods agree well with each other, and that there might be irreducible differences between them and SCOP.

Automatic methods VAST, SHEBA and DALI obtained 71%–76% TPR_01 _using the combination of Ward's method and the MI cut strategy which maximizes the agreement with SCOP. This is higher than the maximum 65% and 67% obtained by the largest size cut and the direct pairwise comparison scheme, respectively, on the same set of the SCOP C class protein domains. As the MI cut strategy proceeds with prior knowledge of SCOP folds information, this range of agreement is close to the maximum obtainable between automatic classification and manually curated SCOP dataset, regardless of the partitioning procedure. The inherent level of disagreement can be seen from the number of SCOP folds for which the spanning cluster exceeds the size of the fold, in Table 6.

Examination of two such folds corroborates the findings of our previous paper [15] that some SCOP folds includes structural variation causing measured similarity between members of the same fold to fall below that between members of related, but different folds. Indeed, the distance matrix in Figure 5 shows that fold c.58 displays this pattern, due to low similarity among domains from its distinct homogeneous clusters, and a high similarity with domains from a different fold c.78. The superpositions in Figure 6 (a), (b) and 6 (c), indicate that in all cases, be it intra or inter-cluster, all three structure comparison methods aligned the structural feature defining the fold, and yet intra-cluster structural similarity is higher than inter-cluster structural similarity. Regarding the TIM barrel fold, a detailed study by Nagano *et *al. [23] also characterized the fold as highly diverse, although using the CATH [24] database. Thus, structural variation within folds, shown in [15] as the main cause of divergence between automatic and expert-curated classifications, does not disappear even after satisfying the partitioning constraint. This strengthens our previous findings and emphasizes the importance of properly handling the structural variation in order to reduce the gap between automatic and expert-curated partitions. We also have underlined some relationships between distinct folds. The domains which correspond to the excess span of a dispersed fold could be seen as evidence suggestive of a evolutionary relationship among folds, as discussed by Lupas *et *al [25]. Domains from fold c.6, which are excess span of TIM barrel fold, are actually variants of TIM barrels.

Finally, in the light of this analysis, we think that future improvements to automatic protein structures classification would likely come by explicitly identifying common structural cores. Pairwise similarity scores alone appear to be limited in that regard, so techniques involving multiple structural alignment will likely be needed.

## Conclusion

The level of agreement between manual and automatic classifications varies with clustering methods and tree partitioning strategies. However, the best agreement reaches similar upper bound than when structural pairwise similarity is compared directly to the manual classification. Therefore divergence between automatic and manual classifications is not eliminated by the introduction of partitioning constraints.

Our observations are based on SCOP C class, but are likely valid for other classes as well, as C class domains contain both types of secondary structure elements, alpha helix and beta strand, and are the most difficult to classify due to higher confusion among them compared to the all alpha or all beta classes [15]. Our exploration of potential classification procedures of proteins based on structural similarity is complementary to the analysis done in our previous paper [15]. The modular structure of the proteins has been accounted for by using a database of structural domains defined in SCOP although this domain parsing may be at variance with other domain parsing such as that in CATH [24], or those based on amino acid sequences in CDD[26], ProDOM [27] or Pfam [28]. We did not address this issue here. Notwithstanding efforts made in this present work, there is still a discrepancy between the results of the automatic structure comparison methods and the SCOP classification. Based on the maximum attainable TPR of about 76%, roughly one quarter of the C class domains are not classified by SCOP according to measured global structural similarity. As we previously suggested, the main reason is that global structure similarity cannot entirely account for the characteristic local structural features on which the SCOP classification is based. Future research should be aimed at finding algorithms able to automatically extract such evolutionarily conserved, common local structural features of domains. As observed by Chothia and Lesk [29], in proteins having low sequence identity and the same biochemical function, only about half of secondary structures are conserved. The remaining challenge is to identify the conserved half!

## Methods

We consider a set *E *of *M = *1330 domains in the C class selected from the set of SCOP domains with less than 40% pairwise sequence identity in ASTRAL [30] version 1.63. Their associated *N *= 94 folds constitute a partition, i.e. a collection of disjoint folds or subsets ${\left\{F_{j}\right\}}_{j=1}^{N}$ of *E *whose union is *E*.

Our objective is to compare this fold partition, an expert-curated classification, with a partition of the same set *E *of domains, obtained automatically as follows.

First, all *M*^2 ^= *1,768,900 *pairs of domains drawn from this set *E *were compared by VAST, SHEBA and DALI. The calculations were made using the high-performance computational capabilities of the Biowulf PC/Linux cluster at the National Institutes of Health, Bethesda, MD [31].

Data resulting from the computation were used to produce matrices of similarity scores [*S*_*qt*_], where *S*_*qt *_is either the *Nres *(*Number of Matched Residues*) between domains *q *and *t*, or the *Zscore *for SHEBA and DALI and the *Pcli *score for VAST.

Second, these similarity matrices were transformed into distance matrices and symmetrized by replacing the upper diagonal values with the lower diagonal values. There exists many ways of obtaining a pseudo-distance measure from a similarity measure [32], even though the triangular inequality is not always guaranteed. We choose a simple approach which guarantees that self-distance is zero and all distances are non-negative. With similarity defined as the number of matched residues, the distance satisfies the triangular inequality. For domains *q *and *t *within *E*, and similarity measure *S*_*qt*_, the distance *D*_*qt *_is defined as follow:

(1) *D*_*qt *_= *S*_*qq *_+ *S*_*tt *_- *2 S*_*qt*_

These matrices of pairwise distances were then entered into hierarchical clustering algorithms to obtain dendrograms or binary trees. We considered Matlab implementation [33] of four representative hierarchical clustering methods [20]: Single, Average, Complete linkage and Ward's method.

### Tree cutting algorithm

A dendrogram resulting from a hierarchical clustering is a binary tree, where each node is associated with an inter-cluster distance defined by the joining distance between its left and right children. The inter-cluster distance for the leaves of the tree is defined to be zero. To obtain a partition from a dendrogram, one ordinarily chooses a level, i.e. an inter-cluster distance value, falling within the dendrogram, then removes or "cuts" all the join-nodes above the chosen level, leaving behind a set of trees whose roots or join-nodes fall below that specified level. Here, we generalize this approach and find partitions made up of a set of sub-trees which join with root nodes at various levels, by introducing a panel of tree cutting criteria making use of a variety of tree characteristics, in addition to the inter-cluster distance. Our recursive algorithm starts with the original tree. When its root is cut or deleted, two trees remain, representing the two clusters in the growing current partition. Depending on the number of clusters desired and various topological features of each of the remaining trees, one tree is selected and its root is cut, leaving behind two smaller trees, each again representing a cluster in the refined partition. The procedure is terminated when the desired stopping criterion is reached.

More formally the tree cutting algorithm starts from the initial partition consisting of one cluster containing all elements of *E*. Given a partition $K=\overset{\left|K\right|}{\underset{i=1}{\cup }}K_{i}$ of size |*K*| > 1, a partition of size |*K*| + 1 is obtained from *K *by splitting one of the clusters *K*_*i *_into 2 distinct clusters. The splitting of a cluster is represented by removing the root node of its associated tree.

At the initial step of this process, there is no choice, but cutting the root node of the tree. Further partitioning involves a choice of which cluster to split. A tree cutting strategy determines this choice. We present below several strategies which have been developed here and used in this analysis. We consider three different ways of stopping the tree partitioning process. First, the partitioning process stops when no further partition can be obtained, corresponding to the situation where all clusters of the partition are singletons. Second, the partitioning process stops when a partition with a given number of clusters is obtained. We will be mainly interested by partitions of size 94 clusters corresponding to the number of SCOP C class folds in the dataset. Third the partitioning process stops based on reaching < = 1% FPR (see below) when comparing the automatic partition with SCOP.

### Tree cutting strategies independent of SCOP

We define seven SCOP independent tree cutting strategies. Such a strategy determines the next sub-tree to cut, without using a prior knowledge of SCOP fold partition. They all have been implemented for this analysis.

#### Level cut

**Figure 8:**
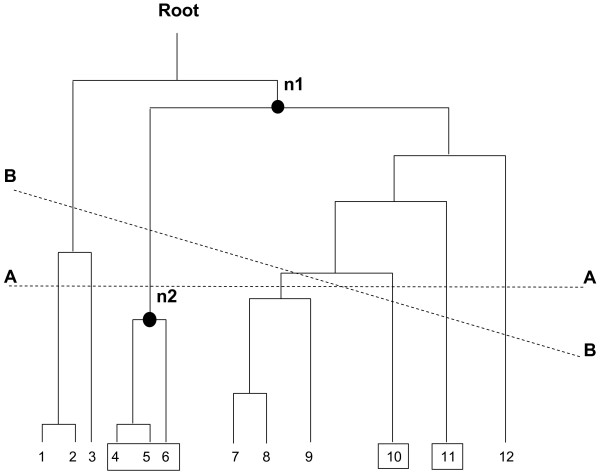
Schematic of cutting strategies and spanning clusters. Leaves of the binary tree, e.g. domains, are numbered 1 to 12. In the figure, it is assumed that four clusters (1,2,3), (7, 8, 9) and (12) are from one particular fold and the three clusters (4, 5, 6), (10) and (11) contain all 5 domains of another fold. Horizontal line A – A represents the level cut, which produces a partition of 7 clusters but which splits the cluster (1,2,3) into two clusters, (1,2) and (3). Oblique line B – B is an allowable cut which produces a partition of 6 clusters and does not split (1,2) from (3). The node n1 represents the spanning cluster of the fold having domains 4, 5, 6, 10 and 11. The span, or size, of this spanning cluster is nine, and its excess span, or number of included domains not in this fold, is four. The node labelled n2, spanning three domains, namely 4, 5, and 6, represents the largest homogeneous cluster of this fold. For this fold, the relative size of the largest homogeneous cluster is 3/5, e.g. the size of the largest homogeneous cluster divided by the size of the associated fold.

The strategy proceeds by descending inter-cluster distance, starting at the level of the root node. At a given step of the tree cutting strategy, resulting trees are available candidates for further cutting. The level of the next cut is determined by the tree whose node has the highest inter-cluster distance among all candidates. An illustration of the level cut is given in Figure 8.

#### Largest size cut

At a given step of the tree cutting strategy, resulting trees are available candidates for cutting. The candidate tree which contains the largest number of domains or leaves, is cut. This strategy counts the exact number of domains in the subtree evaluated for cutting.

#### Highest tree cut

The topological height *h(K*_*i*_*) *of the node *K*_*i *_is the number of intermediate nodes along the longest path from *K*_*i *_to the leaves. The leaves are of topological height zero. For each cluster *K*_*i*_, the topological height of its associated sub-tree is computed. The tree with the topologically highest root node is cut.

#### Tree completeness cut

Given the height *h(K*_*i*_*) *of the sub-tree with root node *K*_*i*_, the number of nodes in this sub-tree if it were complete would be 2^*h*(*Ki*)+1^-1. If the sub-tree is not complete, its actual number of nodes, including the root and the leaves, is less than 2^*h*(*Ki*)+1^-1. Tree completeness is defined as the ratio of the actual number of nodes of the sub-tree, and the number of nodes if it were complete. The smaller the ratio, the less complete is the tree. The tree with the smallest ratio (least complete) is cut.

#### Tree skewness cut

Given a tree, its skewness is defined as the ratio of the number of nodes in its left and right children. The ratio is defined such that the number of nodes of the smallest child, left or right, is divided by the number of nodes of the largest child. The tree of greatest skewness is chosen for cutting.

#### Longest branch cut

The branch length of a tree is the difference between the intercluster distance of the root node and the smaller of the inter-cluster distances of its two children. The tree with the longest branch value is cut.

#### Maximum entropy cut

The entropy *H(K) *of an entire partition *K *is defined by[34]:

(2) $$H(K)=-\sum_{i\in K}P_{i}\ln P_{i}$$

where $P_{i}=\frac{\left|K_{i}\right|}{M}$, the probability of the cluster *K*_*i*_, is ratio of the number of domains within *K*_*i *_to the total number *M *of domains being partitioned. The tree which would result in the greatest increase in entropy value for the current partition is chosen for cutting.

### Tree cutting strategies which refer to SCOP

We define here three tree cutting strategies which use the prior knowledge of SCOP fold partition to determine the next sub-tree to cut:

#### Mutual Information (MI) cut

The mutual information *I(K:F) *between a partition *K *resulting from the cutting of a binary tree and the SCOP fold partition *F*, is defined as follow:

(3) $$I(K:F)=\sum_{i\in K}\sum_{j\in F}P_{ij}\ln\frac{P_{ij}}{P_{i}P_{j}}$$

with *P*_*i *_as defined for entropy, and $P_{ij}=\frac{\left|K_{i}\cap F_{j}\right|}{M}$ is ratio of the number of domains in common to cluster *K*_*i *_and fold *F*_*j *_to *M*. Cutting a particular tree results in a new partition, as well as a new value of *I(K:F)*. The tree which would result in the greatest increase of the mutual information is cut.

#### Best TPR/FPR Ratio cut

TPR and FPR values (defined below) refer to the SCOP fold partition. The tree whose cutting would results in the greatest TPR/FPR ratio, is cut.

#### Best TPR cut

The tree which would result in the greatest TPR value is cut.

### ROC curves for comparing partitions

The ROC curves are constructed using the definition of true and false positive rates introduced in [15], but with the notion of membership in a cluster replacing the notion of similarity cutoff value. The following definitions reflect this variation.

The true positive rate between two partitions is defined as

(4) $$TPR=\frac{1}{M}\sum_{j=1}^{N}n_{j}TPR_{j}$$

where *TPR*_*j *_is defined by:

(5) $$TPR_{j}=\sum_{k}\left(n_{jk}^{2}-n_{jk}\right)/n_{j}(n_{j}-1)$$

where *n*_*jk *_is the number of domains common to cluster *k *and SCOP fold *j*, and *n*_*j *_is the number of domains in fold *j*.

The false positive rate between partitions is defined as follow:

(6) $$FPR=\frac{1}{M}\sum_{j=1}^{N}\frac{n_{j}}{M-n_{j}}\left(\sum_{i=1;i\neq j}^{N}n_{i}FPR_{ij}\right)$$

where

(7) $$\begin{array}{cc} FPR_{ij}=\sum_{k}n_{ki}n_{kj}/n_{i}n_{j}, & \text{for }i\neq j \end{array}$$

when *i *and *j *referring to SCOP folds *i *and *j*.

### Distance between partitions

Given two partitions *K *and *P*, we define the distance between these two partitions as follow:

(8) $$\Delta =\sum_{i}^{M}\sum_{j}^{M}\left|K\left(i,j\right)-P\left(i,j\right)\right|$$

where *K *and *P *are binary incidence matrices of the two partitions whose elements take on the value 1 when the domains i and j are in the same cluster, and 0 otherwise.

### Multidimensional scaling (MDS) plot

We use classical multidimensional scaling to plot a set of points in Euclidean space, such that each point represents a partition and the Euclidean distance between two points in the plot approximates the value of the Δ-distance between partitions they represent. Thirteen partitions are represented in the plot, including the expert-curated fold partition SCOP. Automatic partitions were chosen to have as many clusters as folds in the C class, by requiring the partitioning algorithm to stop when a partition of size 94 clusters is reached. The computation of pairwise Δ-distance between the 13 partitions resulted in a 13 by 13 matrix (see Table 3) which is then used as input to the classical MDS procedure in Matlab [35]. A two dimensional plot of the 13 partitions is obtained by projecting the points onto the two X and Y axes where the X axis is the eigenvector corresponding to the largest positive eigenvalue, and the Y axis the eigenvector corresponding to the second largest eigenvalue.

### Measure of fold dispersion

We define a measure of the dispersion of a SCOP fold within a binary tree, based on two metrics: the size of the excess span of the fold, and the size of the largest homogeneous cluster of the fold. The spanning cluster of a SCOP fold is the smallest cluster in the tree including all its domains. Proceeding upward from the leaves (domains) of that fold and stopping at the first (lowest) node common to all its leaves, we find the spanning cluster of the fold. The size of this spanning cluster, i.e. the number of leaves or domains it includes, is always greater or equal to the size of the associated fold. The set theoretic difference between the spanning cluster and the associated fold, is called excess span. The intersection of spanning clusters from distinct trees formed using distinct methods, identifies domains which might reasonably be considered as similar to domains in that fold. The size of the intersection less the members of that fold is a measure of how much larger the fold should be if it were to include all reasonably similar domains.

A homogeneous cluster is one containing domains of only one fold. Folds may be comprised of more than one homogeneous cluster. The size of the largest homogeneous cluster as a fraction of the size of a fold measures the fraction of the fold which can be easily recognized by the similarity measure and clustering algorithm. Figure 8 illustrates the notion of homogeneous and spanning clusters for a schematic binary tree.

## Authors' contributions

VS, PM – execution of pairwise comparisons using VAST on Biowulf computer, development of partition algorithms, statistical analysis

JFG, JG – development of VAST program, interpretation of results

CHT, BL – development of SHEBA program, and pairwise comparisons, 3D structure visualization, interpretation of results

All authors participated in the formulation of the problem and in devising specific solutions to the problem. All authors have read and approved the final version of the manuscript.
